# Environmental Enteropathy: Elusive but Significant Subclinical Abnormalities in Developing Countries

**DOI:** 10.1016/j.ebiom.2016.07.030

**Published:** 2016-07-26

**Authors:** Koji Watanabe, William A. Petri

**Affiliations:** a Division of Infectious Diseases and International Health, University of Virginia, Charlottesville, VA, USA; b AIDS Clinical Center, National Center for Global Health and Medicine, Tokyo, Japan

**Keywords:** Environmental enteropathy, Environmental enteric dysfunction, Oral vaccine, Growth faltering, Neurocognitive development

## Abstract

Environmental enteropathy/Environmental enteric dysfunction (EE/EED) is a chronic disease of small intestine characterized by gut inflammation and barrier disruption, malabsorption and systemic inflammation in the absence of diarrhea. It is predominantly diseases of children in low income countries and is hypothesized to be caused by continuous exposure to fecally contaminated food, water and fomites. It had not been recognized as a priority health issue because it does not cause overt symptoms and was seen in apparently healthy individuals. However, there is a growing concern of EE/EED because of its impact on longitudinal public health issues, such as growth faltering, oral vaccine low efficacy and poor neurocognitive development. Recent works have provided important clues to unravel its complex pathogenesis, and suggest possible strategies for controlling EE/EED. However, effective diagnostic methods and interventions remain unsettled. Here, we review the existing literature, especially about its pathogenesis, and discuss a solution for children living in the developing world.

## Highlights

• EE/EED is acquired by the close contact with unsanitary condition, and abnormalities of EE/EED are reversible over time.
• EE/EED should be diagnosed as young as possible because poor growth is often evident within 1-2 years of life.
• EE/EED should be diagnosed by simple, noninvasive and low-cost methods, such as fecal biomarkers.
• It is hard to diagnose EE/EED and to predict consequences by a single marker/test due to the complexity in pathogenesis.
• Interventions for EE/EED other than sanitary reform are under investigation although no reports showed favorable outcomes currently.

## 1 Introduction

### 1.1 Importance of EE/EED in developing world

Environmental enteropathy/Environmental enteric dysfunction (EE/EED) was firstly described as “tropical enteropathy” in 1960′s, and was defined by chronic histological changes of small intestinal inflammation in individuals living in tropical areas (Cook et al., 1969, Colwell et al., 1968, Garcia, 1968). Histopathological abnormalities were characterized by decreased villous height, crypt hyperplasia, lymphocytic infiltration of the lamina propria, and increased intraepithelial lymphocytes, which were accompanied by abnormalities in the absorptive function of small intestine, such as xylose and vitamin B12. Since its discovery, tropical enteropathy had not been recognized as a priority health issue because it does not cause overt symptoms and was seen in apparently healthy individuals. However, EE/EED has risen in prominence as a potential cause of growth faltering in children living in developing countries. Also, recent studies demonstrated the association of ED/EED with poor neurocognitive development and low vaccine efficacy (Guerrant et al., 2013, Korpe and Petri, 2012, Lunn et al., 1991, Jiang et al., 2014), resulting in the current great concern of ED/EED in public health of developing countries.

Although the exact pathogenesis of ED/EED and the developmental mechanism of subsequent sequelae remain to be defined, in this review we will share the suggested etiology of ED/EED and discuss a solution for children living in developing countries.

### 1.2 Search strategy and selection criteria of referenced papers in this review

Data for this Review were identified by searches of PubMed, and references from relevant articles using the search terms “environmental enteropathy” and “environmental enteric dysfunction”. Only articles published in English between 1960 and 2016 were included.

### 1.3 Etiology of EE/EED

EE/EED is hypothesized to be caused by the continuous exposure to contaminated fecally contaminated food, water or fomites in poor sanitary condition. However, the etiology of EE/EED had been misunderstood for a while since its discovery that “tropical enteropathy” occurs due to the location where affected individuals are living, such as climate. Geographical data comparing the intestinal permeability among asymptomatic volunteers from 14 different countries showed “Tropical enteropathy” was present across the tropics, but absent in some tropical areas with high socio-economical countries, such as Singapore and Qatar (Menzies et al., 1999), which supports the idea that these abnormal changes are dependent on the socio-economic status and not on the tropical climate. Therefore, in the present day, “tropical enteropathy” is renamed as “Environmental enteropathy (EE)” or “Environmental enteric dysfunction (EED)”.

EE/EED can be acquired by a certain period of time of living in poor sanitation and hygiene, whereas Celiac disease, showing the similarities with EE/EED histologically, occurs among genetically susceptible individuals exposed to gluten containing foods. Early investigations of Pakistanis and visiting residents to Pakistan showed that differences in intestinal permeability and absorptive capacity were related to the area of residence, not the areas of their origin (Lindenbaum, 1968, Lindenbaum et al., 1971), which was later confirmed by the studies performed in West Birmingham (Wood et al., 1991, Iqbal et al., 1996). One study showed that Peace Corps volunteers moving to Pakistan from the United States exhibited malabsorption of xylose (40% of participants) and Vitamin B12 (48%) after 6 months of residence in Pakistan. Moreover, all participants showed abnormalities in histology of jejunal biopsy specimen at that time point. On the other hand, malabsorption of xylose was present in only 3.3% of individuals working for diplomatic and technical assistance organizations who resided in Pakistan during the same period as the Peace Corps group (Lindenbaum, 1968). Another study, which investigated asymptomatic Indians and Pakistanis who had moved to the United States, demonstrated that significant improvement in both xylose absorption and villus architecture occurred with increasing periods of residence in US (Gerson et al., 1971). On the other hand, cross sectional analysis of UK immigrants from India and Afro-Caribbean countries showed that immigrants showed higher gut permeability and abnormal histological findings compared to UK born individuals although this study lacked the data of the time from immigration to the study (Iqbal et al., 1996). These results demonstrated that EE/EED is acquired by the close contact with unsanitary condition, and abnormalities of EE/EED are at least partially reversible over time.

### 1.4 Definition of disease and the difficulties in diagnosis in children

There are no clear diagnostic criteria of EE/EED. Identification of blunted villi and crypt hyperplasia in biopsy specimens collected by upper gastrointestinal endoscopy might be required to support the diagnosis of EE/EED. However, these histological changes, despite the various extents in each individual, are seen in almost all people living in unsanitary conditions in developing countries (Campbell et al., 2003b, Lindenbaum et al., 1966), whereas extent of physical or mental poor development varies widely. Considered together, the presence of abnormalities in biopsy may not be a predictor of poor outcomes from EE/EED, such as growth faltering, oral vaccine failure and poor neurocognitive development. Furthermore, poor growth is often evident within 1–2 years of life and is largely irreversible (Black et al., 2008). It is also known that undernutrition (lower height and weight) at 2 years is associated with lower human capital, represented by shorter adult height, less schooling, reduced economic productivity, and offspring birth weight from the analysis of multiple prospective cohort (Victora et al., 2008). Diagnosis of ED/EED should be made before at least 2 years of age. However, it is too invasive to perform biopsy by upper gastrointestinal endoscopy of young children (< 2 year-old). These results suggest that simple, noninvasive and low-cost method should be developed for the diagnosis of ED/EED in developing countries.

Urinary lactulose to mannitol (L:M) ratio is the most commonly used noninvasive method for the diagnosis of EE/EED in previous studies (Keusch et al., 2013, Denno et al., 2014). In one study, it was shown that L:M ratio is well correlated with histopathological change (intraepithelial cell lymphocyte number) (Campbell et al., 2003b). However, L:M ratio is not standardized among studies. Also, it is technically complicated for infants because fasting before testing and up to five hour collection of urine are required. Shorter duration of urine collection might induce insufficient sampling due to involuntary micturition of infants. On the other hand, longer duration can be associated with test failure of bagged urine collections (Denno et al., 2014).

Biomarkers using stool or blood samples are less invasive diagnostic methods, and previous studies have shown their usefulness for the diagnosis of EE/EED (Wang et al., 2000, Mondal et al., 2012, Donowitz et al., 2016, George et al., 2015, Benzoni et al., 2015, Lin et al., 2013). Recently, Naylor et al. assessed 22 biomarkers of EE/EED by stool or blood from 700 newborn infants who are participants of the PROVIDE (Performance of Rotavirus and Oral vaccines in Developing Countries) study in Bangladesh (Naylor et al., 2015). This study showed that combinations of these biomarkers are useful for the prediction of future growth faltering or oral vaccine low efficacy. They also showed the difficulty to determine a single marker for the diagnosis of ED/EED because of its complexity in pathogenesis.

As described later, EE/EED is not a simple pathological condition of the increased gut permeability by the morphological changes of the gut, but consists of multiple abnormalities, including enteric and systemic inflammations and subsequent metabolic changes. Thus, we should take into account the disease complexity at the diagnosis of EE/EED.

## 2 Pathogenesis of ED/EED

**Fig. 1:**
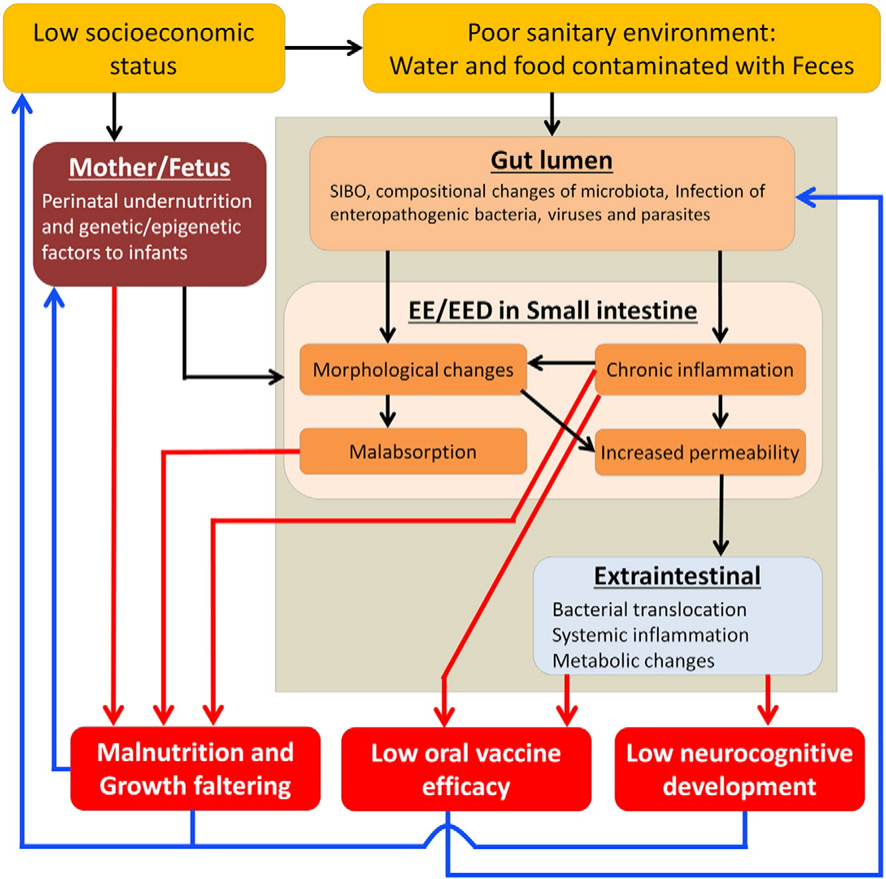
**Pathogenesis of EE/EED and long-lasting consequences.** Continuous exposure to feces triggers EE/EED in individuals living in poor sanitary condition. Pathogenic conditions are seen systemically as well as locally in small intestine (black arrows). Pathogenic conditions have adverse effects on children's health as inducing long-lasting consequences (red arrows). These consequences, in turn, contribute a vicious cycle (blue arrows). Abbreviations, EE, environmental enteropathy; EED, environmental enteric dysfunction; SIBO, small intestine bacterial overgrowth.

The hypothesized pathogenesis of EE/EED is shown in Fig. 1. People living in poor sanitary conditions are continuously exposed to intestinal bacteria, viruses and parasites by ingestion of food and water contaminated with feces. Ingested bacteria may stimulate the small intestine, resulting in the chronic gut inflammation accompanied by morphological changes, such as flattened villi and crypt hyperplasia. Subsequent increased gut permeability, caused by chronic inflammation and morphological changes in small intestine, induces bacterial translocation followed by systemic inflammation and metabolic changes. Malabsorption, which might be explained by flattened villi, is often accompanied with morphological changes of small intestine (Kelly et al., 2016), resulting in the malnutrition. It is assumed that these abnormalities are overlapping but not equitably occurring in each case. We have to realize which abnormality is present in each individual in order to design effective interventions.

### 2.1 Impact of continuous exposure to feces on gut microbiome in the small intestine

Although it is likely that continuous exposure to bacteria from feces in upper gastrointestinal tract is the main cause of EE/EED, based on epidemiological studies, it is still unclear how these enteropathogens trigger the development of EE/EED.

Small intestine bacterial overgrowth (SIBO) is a subclinical quantitative abnormality of bacteria in the upper gastrointestinal tract defined by greater than 10^5^ CFU/mL upper intestinal aspirate as assessed by both anaerobic and aerobic cultures (O'mahony and Shanahan, 2010). Although SIBO is observed among patients with anatomic abnormalities (adhesions after surgery or radiation), inflammation (inflammatory bowel diseases and HIV infection), and metabolic disorders (diabetes mellitus), it is frequently seen in people living in developing countries (Donowitz and Petri, 2015). Importantly, it was also shown that SIBO is associated with growth faltering. An earlier study in Myanmar performing breath hydrogen test after rice meal as a diagnosis of SIBO on 256 village children showed 20.7% of the participants had SIBO and children having SIBO had a high relative risk of having faltered growth (Khin Maung et al., 1992). Donowitz et al. performed glucose hydrogen breath testing for 90 Bangladesh 2-year-old children, and showed a strong association between the presence of SIBO and higher intestinal inflammation markers (fecal REG1beta and fecal calprotectin). They also showed the presence of SIBO is negatively correlated with growth rate from birth to 2 year of age (delta length for age Z score from birth to 2 years of age) (Donowitz et al., 2016). However, in this study, intestinal permeability and systemic inflammation, which are usually elevated in patients with EE/EED, were not associated with the presence of SIBO. These results suggested SIBO partially plays an important role in development of EE/EED.

On the other hand, continuous exposure to fecal material may induce qualitative (compositional) changes of gut microbiota. One report from Bangladesh cohort demonstrated the association between large intestinal microbiota assessed by monthly collected stool samples and severe acute malnutrition. In this study, gut microbiota maturity was measured by “relative microbiota maturity index” and “microbiota-for-age Z-score” which were calculated from the child's fecal microbiota relative to healthy children of similar chronologic age. Gut microbiota immaturity correlated with growth faltering as well as malnutrition (Subramanian et al., 2014). On the other hand, previous studies on Celiac disease have shown the association between compositional changes of microbiota in small intestine and disease severity or response to treatment in celiac disease (Wacklin et al., 2013, Wacklin et al., 2014, Sanchez et al., 2013). One of these studies showed that the patients who are resistant to a long-term gluten-free diet (GFD) with persistent symptoms from celiac disease had a higher relative abundance of *Proteobacteria* and a lower abundance of *Bacteroides* and *Firmicutes* compared to patients without symptoms by GFD (Wacklin et al., 2014). Considered together, it is suggested that not only quantitative abnormalities represented by SIBO but also qualitative (compositional) changes of microbiota in the small intestine play an important role in the development of EE/EED. Further studies will be required for the comprehensive understanding of the effect of bacterial exposure on the development of EE/EED (El Aidy et al., 2015).

### 2.2 Chronic immune activation in the gut

Chronic exposure to contaminated food, water and household environment with feces induces an inflammatory response that may be asymptomatic. There is one study which performed comprehensive histopathological analyses in Gambia. In this study, histopathological examinations of duodenal biopsy specimens including immunohistochemical staining were applied to 38 rural Gambian children (0.5–3 years). These children had a range of nutritional and clinical status, with median weight z score was − 4.6 and 75% with diarrhea. They compared these findings with 19 age matched UK controls. All Gambian children had the findings of EE/EED by histopathology. Although morphological changes were independent of nutritional status, T cell numbers rose and B cell numbers in the gut fell with worsening nutrition. They have also shown that the ratio of regulatory cytokines (TGF-β plus IL-10) over inflammatory cytokines (IFN-γ plus TNF-α) was decreased in malnourished children compared to well-nourished children (Campbell et al., 2003b). This study indicated that chronic T cell mediated inflammatory responses are induced during EE/EED. Recent work using a novel RNA selective isolation procedure from human feces, coupled with high-density whole human transcriptome microarray technology also supported the chronic gut inflammations by T cells in children with EE/EED (Yu et al., 2016).

### 2.3 Bacterial translocation

High permeability of the gut induces bacterial translocation into blood. In the study of rural Gambian infants between 8 and 64 weeks of age, plasma concentrations of endotoxin were elevated and related to both growth faltering and measures of mucosal enteropathy in histopathology. This study also demonstrated that immunoglobulin-G-endotoxin core antibody was negatively correlated with height growth (cm/month) and positively correlated with gut permeability (L:M ratio) (Campbell et al., 2003a). These results indicate that increased gut permeability induces translocation of bacterial components into blood and induces immune stimulation, resulting in growth faltering. Recently, in a murine model, it was reported that the gut vascular barrier (GVB), which controls the translocation of antigens into the bloodstream and prohibits entry of the microbiota, can be disrupted by some pathogenic bacteria. In this study, GVB was disrupted directly by *Salmonella*. Also, they demonstrated that the GVD is modified in Celiac disease patients with elevated serum transaminases probably due to bacterial translocation induced by disrupted gut barrier function (Spadoni et al., 2015). These results suggested that bacterial translocation can be enhanced in a vicious cycle by the disruption of gut barrier and by the exposure to pathogenic bacteria.

## 3 Health problems in developing countries: Long-lasting consequences by EE/EED

### 3.1 Malnutrition and growth faltering

Although strategies of breastfeeding promotion for mother and providing sufficient food for children have a large effect on survival, their effect on stunting is small (Dewey and Adu-Afarwuah, 2008, Bhutta et al., 2008, Fekadu et al., 2015, Alemayehu et al., 2015, Yisak et al., 2015, Asfaw et al., 2015). For all developing countries, it is estimated that 32% and 20% of children (< 5 years old) are under-height (height-for-age Z score < − 2) and under-weight (weight-for-age Z score < − 2) for age (Black et al., 2008). The frequency of enteric infections (diarrheal episodes or positivity of pathogenic gut organisms) in early childhood is proven to be a risk factor of growth faltering (Assis et al., 2005, Black et al., 1984, Black et al., 2008, Checkley et al., 2003, Molbak et al., 1997, Valentiner-Branth et al., 2001, Checkley et al., 1998). Furthermore, it was shown in Gambian cohort in 1991 that the persistent increased gut permeability between 2 and 15 months of age was correlated with growth faltering, regardless of diarrheal episodes (Lunn et al., 1991). These results help us to arrive at the awareness that not only sufficient food and adequate breast feeding but also prevention for chronic gut inflammation by EE/EED are essential to achieve expected growth in children living in developing world. Furthermore, linear growth faltering occurs within the first two years of life, and it is irreversible (Black et al., 2008). Considered together, it is necessary to diagnose EE/EED as early as possible in their life.

### 3.2 Vaccine failure (changes in gut immune responses and systemic inflammation)

It has been shown that many oral vaccines, both live and non-living, are less immunogenic and less protective when administered to individuals in developing countries than those in developed countries (Qadri et al., 2013). The PROVIDE study used several makers of systemic inflammation and gut inflammation early in life, and tested the relation between these markers and vaccine efficacy. Gut inflammation and systemic inflammation were closely related to oral vaccine underperformance whereas systemic inflammation was positively associated with better parenteral vaccine responses (Naylor et al., 2015). This result emphasized that low efficacy of oral vaccine in developing countries are, at least partially, due to chronic gut and systemic inflammation. On the other hand, recent clinical trials of oral polio vaccine efficacy after antibiotic treatment could not demonstrate better OPV efficacy although gut inflammation was improved by the antibiotics. In this study, vaccine efficacy (seroconversion) was negatively correlated with the presence of viral pathogens in stool, especially enteroviruses, adenoviruses and wild rotaviruses (Grassly et al., 2016). This study suggested that direct viral interference of viral pathogens with innate antiviral immune mechanisms is another possible explanation of the poor immunogenicity of oral polio vaccine in children in developing countries.

### 3.3 Neurocognitive development failure

It was shown that enteric parasite infections, such as Giardia (Berkman et al., 2002) and Cryptosporidium (Guerrant et al., 1999), and diarrheal episodes as well as stunting during early life are associated with poor neurocognitive development in later life (Berkman et al., 2002, Niehaus et al., 2002, Guerrant et al., 1999, Mendez and Adair, 1999). Also animal models have shown that a perinatal inflammatory state has an adverse effect on neurocognitive development (Boksa, 2010, Dinel et al., 2014, Bland et al., 2010, Spencer et al., 2011). Systemic inflammation induced by intravenous administration of lipopolysaccharide (LPS) in early life (14 days after birth) altered emotional behavior at adolescence (30 days after birth) and adulthood (90 days after birth) in the murine model. In this study, decreased phosphorylation of the glucocorticoid receptor in the prefrontal cortex of brain was shown in mice treated with LPS (Dinel et al., 2014). These studies suggest that systemic inflammation results in subsequent poor neurocognitive development.

Other than systemic inflammation induced by bacterial translocation, alteration of iron metabolism is an additional possible explanation for poor neurocognitive development from EE/EED. EE/EED has an influence on the production of hepcidin which is one of key modulators of iron homeostasis. Chronic systemic inflammation induces hepcidin release from liver (Silva and Faustino, 2015). Hepcidin acts to block iron efflux from various cell types by binding to the iron transfer ferroportin (Fpn), and down regulating its expression (Tussing-Humphreys et al., 2012). Hepcidin finally induces the accumulation of iron in various cells including neurons, astrocytes and microglia in CNS, resulting in accumulation of intracellular iron and lack of iron availability for important development processes, such as myelination, monoamine synthesis, neuronal metabolic activities and the proper development of neuronal morphology (Oria et al., 2016, Urrutia et al., 2013, Murray-Kolb, 2013). Although plasma iron levels are often decreased in children with EE/EED, iron supplementation is complicated by low efficacy in developing countries and can result in a more proinflammatory state in the intestinal tract, as intestinal iron enhances bacterial proliferation (Jaeggi et al., 2015).

In addition, changes of gut microbiota might be related to neurocognitive disorder. Late-onset autism has been associated with abnormal gut microbiota (Finegold et al., 2002). Microbiota regulation of gut derived 5-HT by affecting biosynthesis of enterochromaffin cells may affect neurodevelopment (Yano et al., 2015). Furthermore, some *in vivo* experiments suggested that microbiota metabolites enhance the production of several neurotransmitters, such as gamma-aminobutyric acid and catecholamine from intestinal epithelial cells (Barrett et al., 2012, Asano et al., 2012). Microbiota-mediated changes in blood brain barrier (BBB) permeability were associated with reduced expression of the tight junction proteins occludin and claudin-5, which regulate barrier function in endothelial tissues (Braniste et al., 2014). Although evidence is only now emerging for the association of EE/EED with cognitive impairment in human (Jiang et al., 2014), it is a possible part of the explanation for the impaired neurocognitive development of children living in poverty (Oria et al., 2016).

### 3.4 Mother's stunting results in physical and mental development of next generation

It has been known that Intrauterine Growth Restriction is more frequently seen in the children of mothers with malnutrition. Also, it was reported that maternal malnutrition assessed by BMI < 18.5 kg/m^2^, height or weight was associated with growth faltering in their children (Mondal et al., 2012, Naylor et al., 2015). An additional maternal contribution may be prenatal infection during pregnancy which causes neurocognitive development failure in a murine model (Boksa, 2010). Moreover, it was shown that epigenetic changes influenced by nutrient intake can be passed from mother to infant. Seasonal differences in diet and DNA methylation in people living in rural Gambia showed that maternal nutritional status during early pregnancy caused persistent epigenetic changes at human metastable epialleles (Dominguez-Salas et al., 2014). Considered together, the mother's nutritional status and health is an important determinant of physical and neurocognitive development in children, suggesting that interventions targeting maternal health should be considered for preventing health problems in children.

## 4 Challenges to understand and prevent EE/EED

### 4.1 Efforts for more understanding of the complex pathogenesis of ED/EED

**Fig. 2:**
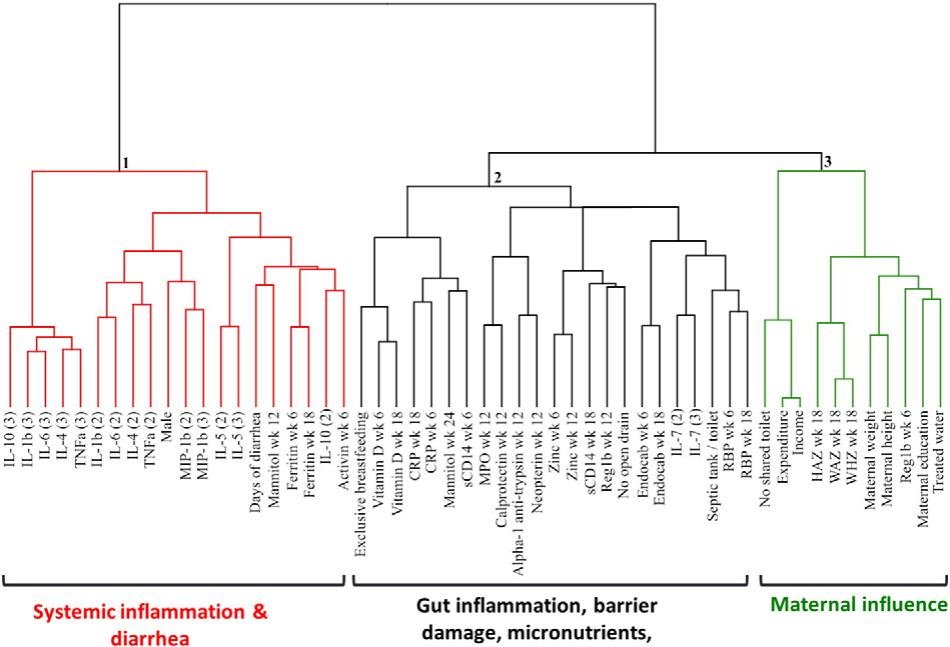
**Cluster dendrogram of biomarkers of EE/EED.** This hierarchical analysis of biomarkers can accommodate a mixture of quantitative and qualitative variables and classify those strongly correlated or similar biomarkers into the same clusters using homogeneity criterion. Adjacent markers are the most closely correlated, while increased distance indicates decreasing correlations. Biological samples (blood or urine) were collected at 6, 12 and/or 18 weeks of age in the PROVIDE study. Cytokines were collected at 18 weeks of age, and analyzed as the association of 50–75th percentile as (2), and 75–100th percentile as (3). Biomarkers were well differentiated by the 3 major clusters, consisting cluster 1 (systemic inflammation in red), cluster 2 (enteric inflammation and malabsorption in black) and cluster 3 (maternal health in green). Adapted from the paper by Naylor et al. (Naylor et al., 2015). Abbreviations, IL, interleukin; TNF, Tissue necrosis factor; MIP, macrophage inflammatory protein; CRP, C-reactive protein; sCD, soluble cluster of differentiation; MPO, myeloperoxidase; Reg, regenerating islet derived protein; RBP, retinol binding protein; HAZ, height-for-age Z-score; WAZ, weight-for-age Z-score; WHZ, weight-for-height Z score.

As described above, EE/EED is complex pathogenic state in which multiple organs, including the small intestine, systemic circulation, liver and brain, are involved sequentially. Although dysfunctions of these organs overlap, the degree of dysfunction may vary among individuals. Moreover, determinant factors of sequelae such as physical and neurocognitive growth faltering and oral vaccine inefficacy, are still undefined. Naylor et al. applied clustering analysis to socioeconomic data, physiological data, and biomarkers measured by blood and urine from Bangladesh children. They demonstrated 3 major clusters by this analysis (Fig. 2). Cluster 1 included days of diarrhea and was dominated by systemic inflammation biomarkers. Cluster 2 contained enteric inflammation and micronutrients. Cluster 3 comprised maternal health markers. They compared the association between each cluster and long-lasting consequences (child growth rate and vaccine efficacy). In this study, each cluster was independently associated with child growth or vaccine efficacy, which emphasized the importance of measuring the degree of dysfunction by each involved organ (or each cluster) in order to precisely predict the risk for later sequelae (Naylor et al., 2015).

The Etiology, Risk factors, and Interactions of Enteric Infections and Malnutrition and the Consequences for Child Health (MAL-ED) study is a prospective longitudinal study established at sites in 8 countries with historically high incidence of diarrheal disease and undernutrition (Kosek et al., 2014). This study is prospectively collecting data of growth, vaccine response and cognitive development as well as gut microbial ecology, enteropathogen infection, and dietary intake. This multinational prospective study is expected to improve scientific understanding of the complex relationships between ED/EED and its long-lasting consequences.

Research on predisposing host factors is also important for developing effective interventions although EE/EED as its name implies may have a predominantly non-genetic pathogenesis. Apolipoprotein E4 (APOE4) is the gene related to cholesterol transport and metabolism. Studies on children living in poor sanitation in Brazil demonstrated that children who having APOE4 have improved cognitive development after heavy diarrheal episodes compared to APOE4 negative children (Oria et al., 2005, Oria et al., 2010), suggesting the existence of a protective host genetic factor against neurocognitive poor development by ED/EED. Further investigation on protective host factors should be encouraged to identify the new diagnostic or treatment strategy.

Other than cohort analyses, we should make efforts for revealing pathogenesis of EE/EED by *in vivo* or *in vitro* experiment. Kelly et al. demonstrated bacterial translocation in patients with EE/EED using confocal laser endomicroscopy (CLE) which is a new technique for the real-time visualization and quantification of intestinal epithelial barrier integrity. They showed cell shedding events, which represent epithelial cell defects assessed by CLE, are positively correlated with plasma LPS concentration (Kelly et al., 2016). Yu et al. reported a novel technique extracting host messenger RNA from stool samples. They collected samples from 259 rural Malawian children with varying states of EE/EED, and identified 12 transcripts associated with the severity of EE/EED, including chemokines that stimulate T-cell proliferation, Fc fragments of multiple immunoglobulin families, interferon-induced mediators that dampen cellular responses to hormones (Yu et al., 2016). These new technologies are expected to further reveal the potential mechanisms of gut damage followed by bacterial translocation in human.

There are some studies using malnourished mice produced by low-protein diet (Clough et al., 2016, Hickman et al., 2014). Maier et al. sought to assess the oral vaccine efficacy among murine protein-energy malnutrition (PEM) model. In this study, PEM mice were produced by the administration of isocaloric multideficient regional basic diet which consists of 5% fat, 7% protein, and 88% carbohydrate. PEM mice showed malnutrition (reduced weight gain) by 3 weeks of age although they could not demonstrate impaired rotavirus vaccine efficacy in these mice (Maier et al., 2013). Also, Brown et al. established a murine EE/EED model treated by early-life consumption of a moderately malnourished diet, in combination with iterative oral exposure to commensal *Bacteroidales species* and *Escherichia coli*. Murine EE/EED model had similar features of human EE/EED, which showed villous blunting in histopathology, higher intestinal permeability, chronic gut inflammation, and subsequent growth faltering (Brown et al., 2015). These novel murine models are expected to add to the understanding of the pathogenesis of ED/EED.

### 4.2 Interventions

Strategies to prevent or treat enteropathogen infections in the gut are one logical approach, although there are no reports which showed favorable outcomes currently. One study assessing seven days treatment with Rifaximin, which is a nonabsorbable and broad-spectrum antibiotic, for children in Malawi did not show an improvement of L:M ratio compared with placebo at 21 days after finishing Rifaximin treatment (Trehan et al., 2009). Azithromycin also did not improve the immunogenicity of oral poliovirus vaccine. In this study, oral 10 mg/kg azithromycin or placebo once daily was given for 3 days, followed by serotype-3 monovalent oral polio vaccine on day 14. However, fecal biomarkers of EE/EED (calprotectin, myeloperoxidase, alpha1-antitrypsin) were reduced in the azithromycin group (Grassly et al., 2016), suggesting that it might need a certain amount of time for recovery of gut immunity from the treatment of EE/EED by antibiotic.

A probiotic trial for 30 days had no effect on L:M ratio of 3–5 years old Malawian children (Galpin et al., 2005). Also, probiotics and prebiotics for severe acute malnutrition (PRONUT study) did not have an impact on the improvement of malnutrition (Kerac et al., 2009).

Other than bacterial targeted strategies, anti-inflammatory agents, which are used for inflammatory bowel diseases or Celiac disease, are potential candidates for treatment of EE/EED (Petri et al., 2014). There is one randomized control trial performed in Nairobi, using Mesalazine. The mechanism of action of Mesalazine is blocking transcription of inflammatory cytokines in colonic epithelial cells (Nielsen and Munck, 2007) which is approved to use for inflammatory bowel diseases (IBD) patients (Jones et al., 2014). In this study, there were no significant differences between the mesalazine and placebo groups in linear growth rate or change in height-for-age z-score after 56 days of study period, which consisted of 28 days treatment followed by 28 days of drug-free period, although primary objective of this study was investigating the safety of mesalazine for children with severe acute malnutrition (SAM). The work of Naylor et al. suggests merit in interventions to improve maternal health and reduce stunting and under-weight at birth to prevent EE/EED (Naylor et al., 2015).

## 5 Conclusions

EE/EED has a great impact on public health in developing countries due to its contribution to poor physical and mental development, and oral vaccine under-performance. Current research has enhanced our understanding of the pathogenesis of EE/EED. In the near future, murine model and new technologies will give us additional insight into its pathogenesis. Non-invasive and cost-effective diagnostic methods which accurately predict the risk of long-lasting poor consequences of EE/EED will be one part of this response. Efforts to improve maternal health and sanitation offer hope for prevention. Understanding EE/EED offers real hope for interventions to allow children in developing world to achieve their full potential.

## References

[bb0005] Alemayehu M., Tinsae F., Haileslassie K., Seid O., Gebregziabher G., Yebyo H. (2015). Undernutrition status and associated factors in under-5 children, in Tigray, Northern Ethiopia. Nutrition.

[bb0010] Asano Y., Hiramoto T., Nishino R., Aiba Y., Kimura T., Yoshihara K., Koga Y., Sudo N. (2012). Critical role of gut microbiota in the production of biologically active, free catecholamines in the gut lumen of mice. Am. J. Physiol. Gastrointest. Liver Physiol..

[bb0015] Asfaw M., Wondaferash M., Taha M., Dube L. (2015). Prevalence of undernutrition and associated factors among children aged between six to fifty nine months in Bule Hora district, South Ethiopia. BMC Public Health.

[bb0020] Assis A.M., Barreto M.L., Santos L.M., Fiaccone R., Da Silva Gomes G.S. (2005). Growth faltering in childhood related to diarrhea: a longitudinal community based study. Eur. J. Clin. Nutr..

[bb0025] Barrett E., Ross R.P., O'Toole P.W., Fitzgerald G.F., Stanton C. (2012). Gamma-aminobutyric acid production by culturable bacteria from the human intestine. J. Appl. Microbiol..

[bb0030] Benzoni N., Korpe P., Thakwalakwa C., Maleta K., Stephenson K., Manary M., Manary M. (2015). Plasma endotoxin core antibody concentration and linear growth are unrelated in rural Malawian children aged 2–5 years. BMC Res. Notes.

[bb0035] Berkman D.S., Lescano A.G., Gilman R.H., Lopez S.L., Black M.M. (2002). Effects of stunting, diarrhoeal disease, and parasitic infection during infancy on cognition in late childhood: a follow-up study. Lancet.

[bb0040] Bhutta Z.A., Ahmed T., Black R.E., Cousens S., Dewey K., Giugliani E., Haider B.A., Kirkwood B., Morris S.S., Sachdev H.P., Shekar M., Maternal & Child Undernutrition Study, G. (2008). What works? Interventions for maternal and child undernutrition and survival. Lancet.

[bb0045] Black R.E., Brown K.H., Becker S. (1984). Effects of diarrhea associated with specific enteropathogens on the growth of children in rural Bangladesh. Pediatrics.

[bb0050] Black R.E., Allen L.H., Bhutta Z.A., Caulfield L.E., De Onis M., Ezzati M., Mathers C., Rivera J., Maternal & Child Undernutrition Study, G. (2008). Maternal and child undernutrition: global and regional exposures and health consequences. Lancet.

[bb0055] Bland S.T., Beckley J.T., Young S., Tsang V., Watkins L.R., Maier S.F., Bilbo S.D. (2010). Enduring consequences of early-life infection on glial and neural cell genesis within cognitive regions of the brain. Brain Behav. Immun..

[bb0060] Boksa P. (2010). Effects of prenatal infection on brain development and behavior: a review of findings from animal models. Brain Behav. Immun..

[bb0065] Braniste V., Al-Asmakh M., Kowal C., Anuar F., Abbaspour A., Toth M., Korecka A., Bakocevic N., Ng L.G., Kundu P., Gulyas B., Halldin C., Hultenby K., Nilsson H., Hebert H., Volpe B.T., Diamond B., Pettersson S. (2014). The gut microbiota influences blood-brain barrier permeability in mice. Sci. Transl. Med..

[bb0070] Brown E.M., Wlodarska M., Willing B.P., Vonaesch P., Han J., Reynolds L.A., Arrieta M.C., Uhrig M., Scholz R., Partida O., Borchers C.H., Sansonetti P.J., Finlay B.B. (2015). Diet and specific microbial exposure trigger features of environmental enteropathy in a novel murine model. Nat. Commun..

[bb0075] Campbell D.I., Elia M., Lunn P.G. (2003). Growth faltering in rural Gambian infants is associated with impaired small intestinal barrier function, leading to endotoxemia and systemic inflammation. J. Nutr..

[bb0080] Campbell D.I., Murch S.H., Elia M., Sullivan P.B., Sanyang M.S., Jobarteh B., Lunn P.G. (2003). Chronic T cell-mediated enteropathy in rural west African children: relationship with nutritional status and small bowel function. Pediatr. Res..

[bb0085] Checkley W., Epstein L.D., Gilman R.H., Black R.E., Cabrera L., Sterling C.R. (1998). Effects of *Cryptosporidium parvum* infection in Peruvian children: growth faltering and subsequent catch-up growth. Am. J. Epidemiol..

[bb0090] Checkley W., Epstein L.D., Gilman R.H., Cabrera L., Black R.E. (2003). Effects of acute diarrhea on linear growth in Peruvian children. Am. J. Epidemiol..

[bb0095] Clough D., Prykhodko O., Raberg L. (2016). Effects of protein malnutrition on tolerance to helminth infection. Biol. Lett..

[bb0100] Colwell E.J., Welsh J.D., Legters L.J., Proctor R.F. (1968). Jejunal morphological characteristics in South Vietnamese residents. JAMA.

[bb0105] Cook G.C., Kajubi S.K., Lee F.D. (1969). Jejunal morphology of the African in Uganda. J. Pathol..

[bb0110] Denno D.M., Vanbuskirk K., Nelson Z.C., Musser C.A., Hay Burgess D.C., Tarr P.I. (2014). Use of the lactulose to mannitol ratio to evaluate childhood environmental enteric dysfunction: a systematic review. Clin. Infect. Dis..

[bb0115] Dewey K.G., Adu-Afarwuah S. (2008). Systematic review of the efficacy and effectiveness of complementary feeding interventions in developing countries. Matern. Child Nutr..

[bb0120] Dinel A.L., Joffre C., Trifilieff P., Aubert A., Foury A., Le Ruyet P., Laye S. (2014). Inflammation early in life is a vulnerability factor for emotional behavior at adolescence and for lipopolysaccharide-induced spatial memory and neurogenesis alteration at adulthood. J. Neuroinflammation.

[bb0125] Dominguez-Salas P., Moore S.E., Baker M.S., Bergen A.W., Cox S.E., Dyer R.A., Fulford A.J., Guan Y., Laritsky E., Silver M.J., Swan G.E., Zeisel S.H., Innis S.M., Waterland R.A., Prentice A.M., Hennig B.J. (2014). Maternal nutrition at conception modulates DNA methylation of human metastable epialleles. Nat. Commun..

[bb0130] Donowitz J.R., Petri W.A. (2015). Pediatric small intestine bacterial overgrowth in low-income countries. Trends Mol. Med..

[bb0135] Donowitz J.R., Haque R., Kirkpatrick B.D., Alam M., Lu M., Kabir M., Kakon S.H., Islam B.Z., Afreen S., Musa A., Khan S.S., Colgate E.R., Carmolli M.P., Ma J.Z., Petri W.A. (2016). Small intestine bacterial overgrowth and environmental enteropathy in Bangladeshi children. mBio.

[bb0140] El Aidy S., Van Den Bogert B., Kleerebezem M. (2015). The small intestine microbiota, nutritional modulation and relevance for health. Curr. Opin. Biotechnol..

[bb0145] Fekadu Y., Mesfin A., Haile D., Stoecker B.J. (2015). Factors associated with nutritional status of infants and young children in Somali Region, Ethiopia: a cross- sectional study. BMC Public Health.

[bb0150] Finegold S.M., Molitoris D., Song Y., Liu C., Vaisanen M.L., Bolte E., Mcteague M., Sandler R., Wexler H., Marlowe E.M., Collins M.D., Lawson P.A., Summanen P., Baysallar M., Tomzynski T.J., Read E., Johnson E., Rolfe R., Nasir P., Shah H., Haake D.A., Manning P., Kaul A. (2002). Gastrointestinal microflora studies in late-onset autism. Clin. Infect. Dis..

[bb0155] Galpin L., Manary M.J., Fleming K., Ou C.N., Ashorn P., Shulman R.J. (2005). Effect of Lactobacillus GG on intestinal integrity in Malawian children at risk of tropical enteropathy. Am. J. Clin. Nutr..

[bb0160] Garcia S. (1968). Malabsorption and malnutrition in Mexico. Am. J. Clin. Nutr..

[bb0165] George C.M., Oldja L., Biswas S., Perin J., Lee G.O., Kosek M., Sack R.B., Ahmed S., Haque R., Parvin T., Azmi I.J., Bhuyian S.I., Talukder K.A., Mohammad S., Faruque A.G. (2015). Geophagy is associated with environmental enteropathy and stunting in children in rural Bangladesh. Am.J.Trop. Med. Hyg..

[bb0170] Gerson C.D., Kent T.H., Saha J.R., Siddiqi N., Lindenbaum J. (1971). Recovery of small-intestinal structure and function after residence in the tropics. II. Studies in Indians and Pakistanis living in New York City. Ann. Intern. Med..

[bb0175] Grassly N.C., Praharaj I., Babji S., Kaliappan S.P., Giri S., Venugopal S., Parker E.P., Abraham A., Muliyil J., Doss S., Raman U., Liu J., Peter J.V., Paranjape M., Jeyapaul S., Balakumar S., Ravikumar J., Srinivasan R., Bahl S., Iturriza-Gomara M., Uhlig H.H., Houpt E.R., John J., Kang G. (2016). The effect of azithromycin on the immunogenicity of oral poliovirus vaccine: a double-blind randomised placebo-controlled trial in seronegative Indian infants. Lancet Infect. Dis..

[bb0180] Guerrant D.I., Moore S.R., Lima A.A., Patrick P.D., Schorling J.B., Guerrant R.L. (1999). Association of early childhood diarrhea and cryptosporidiosis with impaired physical fitness and cognitive function four-seven years later in a poor urban community in Northeast Brazil. Am.J.Trop. Med. Hyg..

[bb0185] Guerrant R.L., Deboer M.D., Moore S.R., Scharf R.J., Lima A.A. (2013). The impoverished gut–a triple burden of diarrhoea, stunting and chronic disease. Nat. Rev. Gastroenterol. Hepatol..

[bb0190] Hickman D., Jones M.K., Zhu S., Kirkpatrick E., Ostrov D.A., Wang X., Ukhanova M., Sun Y., Mai V., Salemi M., Karst S.M. (2014). The effect of malnutrition on norovirus infection. mBio.

[bb0195] Iqbal T.H., Lewis K.O., Gearty J.C., Cooper B.T. (1996). Small intestinal permeability to mannitol and lactulose in the three ethnic groups resident in West Birmingham. Gut.

[bb0200] Jaeggi T., Kortman G.A., Moretti D., Chassard C., Holding P., Dostal A., Boekhorst J., Timmerman H.M., Swinkels D.W., Tjalsma H., Njenga J., Mwangi A., Kvalsvig J., Lacroix C., Zimmermann M.B. (2015). Iron fortification adversely affects the gut microbiome, increases pathogen abundance and induces intestinal inflammation in Kenyan infants. Gut.

[bb0205] Jiang N.M., Tofail F., Moonah S.N., Scharf R.J., Taniuchi M., Ma J.Z., Hamadani J.D., Gurley E.S., Houpt E.R., Azziz-Baumgartner E., Haque R., Petri W.A. (2014). Febrile illness and pro-inflammatory cytokines are associated with lower neurodevelopmental scores in Bangladeshi infants living in poverty. BMC Pediatr..

[bb0210] Jones K.D., Hunten-Kirsch B., Laving A.M., Munyi C.W., Ngari M., Mikusa J., Mulongo M.M., Odera D., Nassir H.S., Timbwa M., Owino M., Fegan G., Murch S.H., Sullivan P.B., Warner J.O., Berkley J.A. (2014). Mesalazine in the initial management of severely acutely malnourished children with environmental enteric dysfunction: a pilot randomized controlled trial. BMC Med..

[bb0215] Kelly P., Besa E., Zyambo K., Louis-Auguste J., Lees J., Banda T., Soko R., Banda R., Amadi B., Watson A. (2016). Endomicroscopic and transcriptomic analysis of impaired barrier function and malabsorption in environmental enteropathy. PLoS Negl. Trop. Dis..

[bb0220] Kerac M., Bunn J., Seal A., Thindwa M., Tomkins A., Sadler K., Bahwere P., Collins S. (2009). Probiotics and prebiotics for severe acute malnutrition (PRONUT study): a double-blind efficacy randomised controlled trial in Malawi. Lancet.

[bb0225] Keusch G.T., Rosenberg I.H., Denno D.M., Duggan C., Guerrant R.L., Lavery J.V., Tarr P.I., Ward H.D., Black R.E., Nataro J.P., Ryan E.T., Bhutta Z.A., Coovadia H., Lima A., Ramakrishna B., Zaidi A.K., Burgess D.C., Brewer T. (2013). Implications of acquired environmental enteric dysfunction for growth and stunting in infants and children living in low- and middle-income countries. Food Nutr. Bull..

[bb0230] Khin Maung U., Bolin T.D., Duncombe V.M., Myo K., Nyunt Nyunt W., Pereira S.P., Linklater J.M. (1992). Epidemiology of small bowel bacterial overgrowth and rice carbohydrate malabsorption in Burmese (Myanmar) village children. Am.J.Trop. Med. Hyg..

[bb0235] Korpe P.S., Petri W.A. (2012). Environmental enteropathy: critical implications of a poorly understood condition. Trends Mol. Med..

[bb0240] Kosek M., Guerrant R.L., Kang G., Bhutta Z., Yori P.P., Gratz J., Gottlieb M., Lang D., Lee G., Haque R., Mason C.J., Ahmed T., Lima A., Petri W.A., Houpt E., Olortegui M.P., Seidman J.C., Mduma E., Samie A., Babji S., Investigators M.-E.N. (2014). Assessment of environmental enteropathy in the MAL-ED cohort study: theoretical and analytic framework. Clin. Infect. Dis..

[bb0245] Lin A., Arnold B.F., Afreen S., Goto R., Huda T.M., Haque R., Raqib R., Unicomb L., Ahmed T., Colford J.M., Luby S.P. (2013). Household environmental conditions are associated with enteropathy and impaired growth in rural Bangladesh. Am. J. Trop. Med. Hyg..

[bb0250] Lindenbaum J. (1968). Small intestine dysfunction in Pakistanis and Americans resident in Pakistan. Am. J. Clin. Nutr..

[bb0255] Lindenbaum J., Alam A.K., Kent T.H. (1966). Subclinical small-intestinal disease in East Pakistan. Br. Med. J..

[bb0260] Lindenbaum J., Gerson C.D., Kent T.H. (1971). Recovery of small-intestinal structure and function after residence in the tropics. I. Studies in Peace Corps volunteers. Ann. Intern. Med..

[bb0265] Lunn P.G., Northrop-Clewes C.A., Downes R.M. (1991). Intestinal permeability, mucosal injury, and growth faltering in Gambian infants. Lancet.

[bb0270] Maier E.A., Weage K.J., Guedes M.M., Denson L.A., Mcneal M.M., Bernstein D.I., Moore S.R. (2013). Protein-energy malnutrition alters IgA responses to rotavirus vaccination and infection but does not impair vaccine efficacy in mice. Vaccine.

[bb0275] Mendez M.A., Adair L.S. (1999). Severity and timing of stunting in the first two years of life affect performance on cognitive tests in late childhood. J. Nutr..

[bb0280] Menzies I.S., Zuckerman M.J., Nukajam W.S., Somasundaram S.G., Murphy B., Jenkins A.P., Crane R.S., Gregory G.G. (1999). Geography of intestinal permeability and absorption. Gut.

[bb0285] Molbak K., Jensen H., Ingholt L., Aaby P. (1997). Risk factors for diarrheal disease incidence in early childhood: a community cohort study from Guinea-Bissau. Am. J. Epidemiol..

[bb0290] Mondal D., Minak J., Alam M., Liu Y., Dai J., Korpe P., Liu L., Haque R., Petri W.A. (2012). Contribution of enteric infection, altered intestinal barrier function, and maternal malnutrition to infant malnutrition in Bangladesh. Clin. Infect. Dis..

[bb0295] Murray-Kolb L.E. (2013). Iron and brain functions. Curr. Opin. Clin. Nutr. Metab. Care.

[bb0300] Naylor C., Lu M., Haque R., Mondal D., Buonomo E., Nayak U., Mychaleckyj J.C., Kirkpatrick B., Colgate R., Carmolli M., Dickson D., Van Der Klis F., Weldon W., Steven Oberste M., Teams P.S., Ma J.Z., Petri W.A. (2015). Environmental enteropathy, oral vaccine failure and growth faltering in infants in Bangladesh. EBioMedicine.

[bb0305] Niehaus M.D., Moore S.R., Patrick P.D., Derr L.L., Lorntz B., Lima A.A., Guerrant R.L. (2002). Early childhood diarrhea is associated with diminished cognitive function 4 to 7 years later in children in a northeast Brazilian shantytown. Am.J.Trop. Med. Hyg..

[bb0310] Nielsen O.H., Munck L.K. (2007). Drug insight: aminosalicylates for the treatment of IBD. Nat. Clin. Pract. Gastroenterol. Hepatol..

[bb0315] O'mahony S., Shanahan F. (2010). Enteric Microbiota and Small Intestinal Bacterial Overgrowth. Sleisenger and Fordrtan's Gastrointestinal and Liver Disease: Pathophysiology/Diagnosis/Management.

[bb0320] Oria R.B., Patrick P.D., Zhang H., Lorntz B., De Castro Costa C.M., Brito G.A., Barrett L.J., Lima A.A., Guerrant R.L. (2005). APOE4 protects the cognitive development in children with heavy diarrhea burdens in Northeast Brazil. Pediatr. Res..

[bb0325] Oria R.B., Patrick P.D., Oria M.O., Lorntz B., Thompson M.R., Azevedo O.G., Lobo R.N., Pinkerton R.F., Guerrant R.L., Lima A.A. (2010). ApoE polymorphisms and diarrheal outcomes in Brazilian shanty town children. Braz. J. Med. Biol. Res..

[bb0330] Oria R.B., Murray-Kolb L.E., Scharf R.J., Pendergast L.L., Lang D.R., Kolling G.L., Guerrant R.L. (2016). Early-life enteric infections: relation between chronic systemic inflammation and poor cognition in children. Nutr. Rev..

[bb0335] Petri W.A., Naylor C., Haque R. (2014). Environmental enteropathy and malnutrition: do we know enough to intervene?. BMC Med..

[bb0340] Qadri F., Bhuiyan T.R., Sack D.A., Svennerholm A.M. (2013). Immune responses and protection in children in developing countries induced by oral vaccines. Vaccine.

[bb0345] Sanchez E., Donat E., Ribes-Koninckx C., Fernandez-Murga M.L., Sanz Y. (2013). Duodenal-mucosal bacteria associated with celiac disease in children. Appl. Environ. Microbiol..

[bb0350] Silva B., Faustino P. (2015). An overview of molecular basis of iron metabolism regulation and the associated pathologies. Biochim. Biophys. Acta.

[bb0355] Spadoni I., Zagato E., Bertocchi A., Paolinelli R., Hot E., Di Sabatino A., Caprioli F., Bottiglieri L., Oldani A., Viale G., Penna G., Dejana E., Rescigno M. (2015). A gut-vascular barrier controls the systemic dissemination of bacteria. Science.

[bb0360] Spencer S.J., Galic M.A., Pittman Q.J. (2011). Neonatal programming of innate immune function. Am. J. Physiol. Endocrinol. Metab..

[bb0365] Subramanian S., Huq S., Yatsunenko T., Haque R., Mahfuz M., Alam M.A., Benezra A., Destefano J., Meier M.F., Muegge B.D., Barratt M.J., Vanarendonk L.G., Zhang Q., Province M.A., Petri W.A., Ahmed T., Gordon J.I. (2014). Persistent gut microbiota immaturity in malnourished Bangladeshi children. Nature.

[bb0370] Trehan I., Shulman R.J., Ou C.N., Maleta K., Manary M.J. (2009). A randomized, double-blind, placebo-controlled trial of rifaximin, a nonabsorbable antibiotic, in the treatment of tropical enteropathy. Am. J. Gastroenterol..

[bb0375] Tussing-Humphreys L., Pusatcioglu C., Nemeth E., Braunschweig C. (2012). Rethinking iron regulation and assessment in iron deficiency, anemia of chronic disease, and obesity: introducing hepcidin. J. Acad. Nutr. Diet..

[bb0380] Urrutia P., Aguirre P., Esparza A., Tapia V., Mena N.P., Arredondo M., Gonzalez-Billault C., Nunez M.T. (2013). Inflammation alters the expression of DMT1, FPN1 and hepcidin, and it causes iron accumulation in central nervous system cells. J. Neurochem..

[bb0385] Valentiner-Branth P., Steinsland H., Santos G., Perch M., Begtrup K., Bhan M.K., Dias F., Aaby P., Sommerfelt H., Molbak K. (2001). Community-based controlled trial of dietary management of children with persistent diarrhea: sustained beneficial effect on ponderal and linear growth. Am. J. Clin. Nutr..

[bb0390] Victora C.G., Adair L., Fall C., Hallal P.C., Martorell R., Richter L., Sachdev H.S., Maternal & Child Undernutrition Study, G (2008). Maternal and child undernutrition: consequences for adult health and human capital. Lancet.

[bb0395] Wacklin P., Kaukinen K., Tuovinen E., Collin P., Lindfors K., Partanen J., Maki M., Matto J. (2013). The duodenal microbiota composition of adult celiac disease patients is associated with the clinical manifestation of the disease. Inflamm. Bowel Dis..

[bb0400] Wacklin P., Laurikka P., Lindfors K., Collin P., Salmi T., Lahdeaho M.L., Saavalainen P., Maki M., Matto J., Kurppa K., Kaukinen K. (2014). Altered duodenal microbiota composition in celiac disease patients suffering from persistent symptoms on a long-term gluten-free diet. Am. J. Gastroenterol..

[bb0405] Wang W., Uzzau S., Goldblum S.E., Fasano A. (2000). Human zonulin, a potential modulator of intestinal tight junctions. J. Cell Sci..

[bb0410] Wood G.M., Gearty J.C., Cooper B.T. (1991). Small bowel morphology in British Indian and Afro-Caribbean subjects: evidence of tropical enteropathy. Gut.

[bb0415] Yano J.M., Yu K., Donaldson G.P., Shastri G.G., Ann P., Ma L., Nagler C.R., Ismagilov R.F., Mazmanian S.K., Hsiao E.Y. (2015). Indigenous bacteria from the gut microbiota regulate host serotonin biosynthesis. Cell.

[bb0420] Yisak H., Gobena T., Mesfin F. (2015). Prevalence and risk factors for under nutrition among children under five at Haramaya district, Eastern Ethiopia. BMC Pediatr..

[bb0425] Yu J., Ordiz M.I., Stauber J., Shaikh N., Trehan I., Barnell E., Head R.D., Maleta K., Tarr P.I., Manary M.J. (2016). Environmental enteric dysfunction includes a broad spectrum of inflammatory responses and epithelial repair processes. Cell. Mol. Gastroenterol. Hepatol..

